# Neurologic Complications of Babesiosis, United States, 2011–2021

**DOI:** 10.3201/eid2906.221890

**Published:** 2023-06

**Authors:** Sara Locke, Jane O’Bryan, Adeel S. Zubair, Melissa Rethana, Anne Spichler Moffarah, Peter J. Krause, Shelli F. Farhadian

**Affiliations:** Yale School of Medicine, New Haven, Connecticut, USA (S. Locke, J. O’Bryan, A.S. Zubair, M. Rethana, A. Spichler Moffarah, P.J. Krause, S.F. Farhadian);; Frank H. Netter MD School of Medicine at Quinnipiac University, North Haven, Connecticut, USA (J. O’Bryan);; Yale School of Public Health, New Haven (P.J. Krause, S.F. Farhadian).

**Keywords:** *Babesia microti*, babesiosis, neurology, parasites, United States

## Abstract

Babesiosis is a globally distributed parasitic infection caused by intraerythrocytic protozoa. The full spectrum of neurologic symptoms, the underlying neuropathophysiology, and neurologic risk factors are poorly understood. Our study sought to describe the type and frequency of neurologic complications of babesiosis in a group of hospitalized patients and assess risk factors that might predispose patients to neurologic complications. We reviewed medical records of adult patients who were admitted to Yale-New Haven Hospital, New Haven, Connecticut, USA, during January 2011–October 2021 with laboratory-confirmed babesiosis. More than half of the 163 patients experienced >1 neurologic symptoms during their hospital admissions. The most frequent symptoms were headache, confusion/delirium, and impaired consciousness. Neurologic symptoms were associated with high-grade parasitemia, renal failure, and history of diabetes mellitus. Clinicians working in endemic areas should recognize the range of symptoms associated with babesiosis, including neurologic.

Babesiosis is an emerging parasitic infection with global distribution. The infection is caused by intraerythrocytic protozoa of the genus *Babesia*. During the past 2 decades, the incidence of babesiosis has increased, particularly in the northeastern and northern midwestern United States. The Centers for Disease Control and Prevention reported an increased babesiosis incidence in Connecticut, USA, from 2011 (2.1 cases/100,000 persons) to 2019 (9 cases/100,000 persons), more than 10 times the incidence reported nationally during that time period (*1*). More than 100 species of *Babesia* have been described in wild and domestic animals. The predominant species causing human disease in the United States is *B. microti* (*1*–*3*). The disease is transmitted primarily through the bite of an infected ixodid tick, which is capable of transmitting several pathogens at the same time, including *Borrelia burgdorferi*, the cause of Lyme disease (*2*–*5*). Babesiosis is less commonly transmitted via blood transfusion, organ transplantation, or through the placenta (*2*,*3*,*6*).

Although most persons with babesiosis experience nonspecific influenza-like symptoms, more severe and prolonged disease can occur in persons >50 years of age; those who are immunocompromised due to asplenia, cancer, or HIV/AIDS or who are receiving immunosuppressive drugs; and those who have chronic heart, lung, renal, or liver disease (*2*,*7*,*8*). Severe infection is associated with high-grade parasitemia and organ failure (e.g., acute respiratory distress syndrome, congestive heart failure, severe hemolytic anemia, or renal failure) and death (*2*,*8*–*11*). Little has been published about babesiosis-induced central nervous system dysfunction (*12*,*13*). Neurologic complications include headache, syncope, neuropathy, retinal nerve infarcts, and altered state of consciousness (*9*,*12*,*14*–*20*). The full spectrum of neurologic complications and underlying pathophysiology are poorly understood, as are factors that predispose patients to neurologic complications.

We conducted this study to investigate the type and frequency of neurologic complications of babesiosis in a group of hospitalized patients and to assess risk factors that predispose patients to neurologic complications. We hypothesized that patients with a diagnosis of babesiosis commonly experience neurologic system manifestations and that those symptoms are most frequent in patients with severe babesiosis. Accordingly, we conducted a retrospective medical record review of all adult patients admitted to Yale-New Haven Hospital (YNHH) in New Haven, Connecticut, USA, during 2011–2021 with laboratory-confirmed babesiosis.

## Methods

### Design and Setting

The sample population included all adult patients (≥18 years of age) admitted to YNHH during January 2011–October 2021 with a diagnosis of babesiosis. Patients were required to have *Babesia* parasites present on thin blood smear or amplification of *B. microti* DNA by PCR to be included. Eligible patients were identified in collaboration with the Yale Center for Clinical Investigation Joint Data Analytics Team.

### Data Collection

We obtained approval for study procedures from the Yale University Human Investigation Committee before data collection (HIC protocol #2000030420). We performed comprehensive medical record reviews and systematically abstracted study variables by using a standardized medical questionnaire (*21*). We recorded demographic and clinical variables, including the following underlying conditions and comorbidities: chronic cardiac, pulmonary, renal, or hepatic conditions; dementia; diabetes mellitus; hypertension; malignancy; migraine; seizure; stroke; and immunocompromised status.

We recorded all neurologic symptoms and complications documented at the time of hospital admission: acute cerebrovascular disease, acute syncope, ataxia/gait disturbance, confusion/delirium, facial droop, focal weakness, headache, impaired consciousness, nerve pain, tremor, language deficit, vision impairment, vertigo, and seizure. The neurologic symptoms and complications we recorded were then independently confirmed by 2 neurologists. We defined confusion/delirium as a deficit in mental status characterized by disorientation, bewilderment, or difficulty following commands (*22*). We categorized impaired consciousness (i.e., diminished arousal and response to stimulation) on the basis of severity using the Glasgow Coma Scale, classifying symptoms as either lethargy, obtundation, stupor, or coma (*23*). Lethargy is a mild reduction in alertness, obtundation is a moderate reduction in alertness, stupor is a condition of deep sleep in which the patient can only be aroused by vigorous external stimulation, and coma refers to a complete lack of motor response to any stimuli from the external environment (*23*,*24*).

We abstracted key laboratory variables related to the severity of infection. We computed median laboratory values and interquartile ranges (IQRs) on the basis of either the recorded minimum or maximum laboratory value, as appropriate. Laboratory parameters included peak parasitemia, minimum hematocrit and platelet count, and maximum blood urea nitrogen, creatinine, aspartate aminotransferase and alanine aminotransferase. We calculated glomerular filtration rate by using the 2021 Chronic Kidney Disease Epidemiology Collaboration equation (*25*).

### Statistical Analysis

We performed statistical analyses by using SAS Studio 3.8 (SAS Institute Inc.). We summarized demographic characteristics of the sample by using appropriate descriptive statistics. We categorized patients according to peak parasitemia (group 1, <1.0%; group 2, 1.0% −10.0%; group 3, >10.0%) (Table 1). For patients with peak thin blood smear results reported as <1%, we used a value of 0.9% in calculations. We compared those subgroups to determine if neurologic manifestations were more frequent in patients with high parasitemia. Given the nonparametric distribution of those data, we reported medians and IQRs for hematologic, hepatic, and renal function laboratory tests. We used Wilcoxon 2-sample tests to compare laboratory results among patients with and without the most common neurologic symptoms.

**Table 1:** Neurologic symptoms during hospital admissions for babesiosis in patients admitted to Yale-New Haven Hospital, New Haven, Connecticut, USA, January 2011–October 2021*

Neurologic symptom	No. (%) patients	Peak parasitemia, no. (%) patients			p value
		<1.0%, n = 45	1.0%–10.0%, n = 81	>10.0%, n = 37	
Headache	52 (31.9)	14 (31.1)	28 (34.6)	10 (27.0)	0.711
Confusion/delirium	27 (16.6)	2 (4.4)	13 (16.1)	12 (32.4)	**0.003**
Impaired consciousness	24 (14.7)	4 (8.9)	9 (11.1)	11 (29.7)	**0.018**
Ataxia/gait disorder	17 (10.4)	3 (6.7)	10 (12.4)	4 (10.8)	0.632
Vision impairment	10 (6.1)	5 (11.1)	4 (4.9)	1 (2.7)	0.266
Acute syncope	6 (3.7)	2 (4.4)	4 (4.9)	0 (0.0)	0.482
Language deficit	5 (3.1)	0 (0.0)	5 (6.2)	0 (0.0)	0.106
Nerve pain	4 (2.5)	1 (2.2)	2 (2.5)	1 (2.7)	1.000
Focal weakness	3 (1.8)	1 (2.2)	1 (1.2)	1 (2.7)	0.794
Tremor	3 (1.8)	1 (2.2)	2 (2.5)	0 (0.0)	1.000
Seizure	2 (1.3)	1 (2.2)	0 (0.0)	1 (2.7)	0.252

*Total sample (n = 163); Column percentages do not sum to 100% due to many patients being affected by multiple neurologic conditions throughout hospitalization. Boldface type indicates statistical significance.

To represent the distribution density of neurologic symptoms by peak parasitemia, we used Prism 9 software (GraphPad Software) to generate violin plots. We analyzed the relationship between peak parasitemia and each reported neurologic symptom by using Mann-Whitney U tests for continuous variables.

We used univariate logistic regression models to test associations between comorbid conditions and the 3 most common neurologic symptoms: headache, confusion/delirium, and impaired consciousness. We noted variables that were significant in unadjusted univariate analysis and entered them into a multivariate model for each neurologic symptom. We set the significance level for univariate and multivariate analysis to p<0.05.

## Results

### Demographic and Clinical Characteristics

We identified a total of 163 hospitalized patients with laboratory-confirmed babesiosis during the study period (January 2011–October 2021**)**. The median age was 67 years (IQR 45–89; range 30–93). Most patients were male (n = 104 [63.8%]). The study population was predominately White or Caucasian (n = 118 [74.7%]). Most patients (n = 160 [98.2%]) were diagnosed with babesiosis by a blood smear positive for intra-erythrocytic organisms that were consistent with *Babesia*. Three patients were diagnosed with babesiosis on the basis of a positive PCR blood specimen in the context of an appropriate acute clinical syndrome. Most patients (n = 117 [71.8%]) had >1 medical comorbidity at admission. The most common comorbidities were hypertension (n = 82 [50.3%]), immunodeficiency (n = 40 [24.5%]), and cardiac disorder (n = 39 [23.9%]). Most of the immunodeficient patients (n = 21 [52.5%]) were asplenic. Other comorbid conditions included diabetes (n = 23 [14.1%]), stroke (n = 13 [8.0%]), chronic kidney disease (n = 8 [4.9%]), migraine (n = 4 [2.5%]), dementia (n = 3 [1.84%]), and seizure disorder (n = 2 [1.2%]). Twelve patients (7.4%) had erythema migrans rash, indicating active co-infection with Lyme disease.

### Neurologic Symptoms of Babesiosis

We recorded the frequency of neurologic symptoms experienced by patients admitted to YNHH for babesiosis (Table 1). More than half (n = 97 [59.5%]) experienced >1 neurologic symptoms. The most frequent symptoms were headache (n = 52 [31.9%]), confusion/delirium (n = 27 [16.6%]), impaired consciousness (n = 24 [14.7%]), ataxia/gait disorder (n = 17 [10.4%]), and vision impairment (n = 10 [6.1%]). Patients noted to have impaired consciousness were 20 (12.3%) classified as lethargic, 3 (1.8%) as obtunded, and 1 (0.6%) as stuporous.

#### Confusion/Delirium and Parasitemia

Although we found no association between the degree of parasitemia and the presence of any neurologic symptoms, patients with confusion/delirium and impaired consciousness were significantly more likely to have a higher peak parasitemia than those without these symptoms (Figure). All 163 hospitalized patients were stratified by peak parasitemia into 3 groups: peak parasitemia of <1% (n = 45), peak parasitemia of 1.0%–10% (n = 81), and peak parasitemia of >10% (n = 37). The 27 patients with confusion/delirium were also stratified into the peak parasitemia groups. Among the 45 patients with a peak parasitemia <1%, 2 (4.4%) patients experienced confusion/delirium. Among the 81 patients with a peak parasitemia of 1.0%–10%, 13 (16.1%) experienced confusion/delirium. Among the 37 patients with a peak parasitemia of >10%, 12 (32.4%) experienced confusion/delirium (Table 1). The median peak parasitemia of the patients who experienced confusion/delirium was significantly higher than that of the patients who did not experience confusion/delirium (p = 0.001) (Figure). Those findings indicate that as parasitemia increases, so does the prevalence of confusion/delirium.

**Figure:**
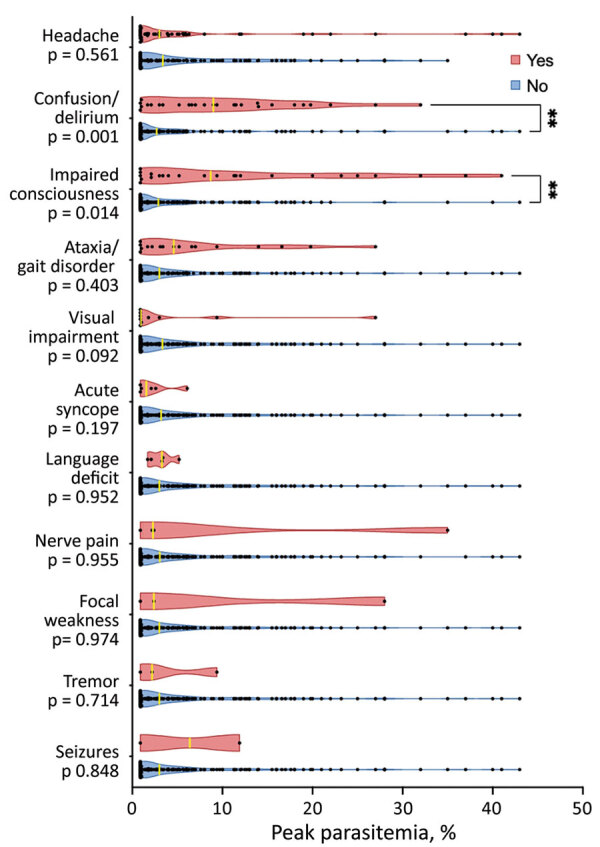
Violin plot depicting the peak parasitemia distribution in patients with and without select neurologic symptoms associated with babesiosis admitted to Yale-New Haven Hospital, New Haven, Connecticut, USA, January 2011–October 2021. Asterisks (**) indicate that the median peak parasitemia significantly differed between the patients who did and the patients who did not experience the specific symptom (p<0.05).

#### Impaired Consciousness and Parasitemia

We found a significant association between peak parasitemia and impaired consciousness (p<0.005). Of the 163 hospitalized patients, 14.7% experienced impaired consciousness at the time of hospital admission. When we stratified by peak parasitemia groups, we observed impaired consciousness in 4 (8.9%) of the group with the lowest (<1%) peak parasitemia, 9 (11.1%) of the group with mid (1.0%–10%) peak parasitemia, and 11 (29.7%) of those in the group with the highest (>10%) peak parasitemia. That distribution was similar to that of patients with confusion/delirium. Patients with impaired consciousness (of any degree) had a higher median peak parasitemia than those without impaired consciousness (p = 0.014) (Figure).

#### Ophthalmologic Symptoms

Ten (6.1%) patients reported transient vision impairment, one of whom was formally evaluated by the ophthalmology department after reportedly seeing different colored lights and shapes when she closed her eyes. Ophthalmologic examination revealed no evidence of ocular infection or inflammation.

### Neuroimaging Findings

Thirty-three (20.2%) of the hospitalized patients underwent neuroimaging with cranial computerized tomography (n = 28), brain magnetic resonance imaging (n = 7), or electroencephalogram (n = 2) (Table 2). Only 2 patients had acute abnormalities seen on neuroimaging, 1 with ischemic stroke and 1 with subarachnoid hemorrhage. The patient who suffered a stroke had fever, malaise, and anemia; an episode of expressive aphasia during an acute babesiosis episode resulting from a small, left-middle cerebral artery stroke, confirmed on imaging to be secondary to high-grade internal carotid artery stenosis. The patient with a subarachnoid hemorrhage had confusion and jaundice and was in critical condition because of multiorgan failure, including respiratory, kidney, and liver failure, and disseminated intravascular coagulation. Computed tomography revealed subarachnoid hemorrhage, most conspicuous within the left frontal lobe. Most other patients who underwent neuroimaging had nonspecific white matter changes and some element of volume loss, which were considered to be related to chronic disease and aging. Electroencephalograms results were unremarkable for both patients who underwent this examination.

**Table 2:** Imaging indications and findings in patients with neurologic symptoms associated with babesiosis admitted to Yale-New Haven Hospital, New Haven, Connecticut, USA, January 2011–October 2021*

Imaging	No. (%) patients
Neuroimaging modality	
Computed tomography	28 (17.2)
Magnetic resonance imaging	7 (4.3)
Electroencephalogram	2 (1.2)
Indication	
Altered mental status/confusion	15 (9.2)
Headache	8 (4.9)
Fever	5 (3.1)
Evaluate CNS abnormalities	3 (1.8)
Weakness	3 (1.8)
Dizziness	1 (0.6)
Dysphagia, slurred speech	1 (0.6)
Fall	1 (0.6)
Head injury	1 (0.6)
Visual changes	1 (0.6)
Syncope	1 (0.6)
Numbness	1 (0.6)
Fatigue	1 (0.6)
Seizure	1 (0.6)
Findings	
Nonspecific white matter changes	23 (14.1)
Volume loss	14 (8.6)
Acute changes	2 (1.2)
Evidence of previous stroke	3 (1.8)

*Total sample consisted of 163 patients. Neuroimaging modalities and indications sum to >33 because some patients received multiple imaging modalities or had multiple indications. CNS, central nervous system.

### Laboratory Findings

We assessed laboratory measures of hematologic, hepatic, and renal function among patients with the most frequently reported neurologic symptoms: headache, confusion/delirium, and impaired consciousness. Anemia was common in the overall sample population. Median hematocrit was 26.7% for male patients (reference range 40%–52%) and 25% for female patients (reference range 37%–53%). Patients with impaired consciousness had a lower hematocrit than did those without impaired consciousness (23.6% vs. 26.4%; p = 0.024) (Table 3). Patients with confusion/delirium and those with impaired consciousness had significantly higher median blood urea nitrogen and creatinine values and significantly lower glomerular filtration rate compared with patients without these symptoms (Table 3).

**Table 3:** Laboratory result comparisons by neurologic symptoms in patients with babesiosis admitted to Yale-New Haven Hospital, New Haven, Connecticut, USA, January 2011–October 2021*

Laboratory result, median (IQR)	Reference range	Headache				Confusion/delirium				Impaired consciousness		
		Yes, n = 52	No, n = 111	p value†		Yes, n = 27	No, n = 136	p value†		Yes, n = 24	No, n = 139	p value†
Hematologic function												
Lowest hematocrit	M, 40%–52%; F, 37%–53%	25.9 (16.2–35.6)	26.0 (19.4­–32.6)	0.581		24.0 (16.5–31.5)	26.4 (18.8–34)	0.088		23.6 (15.7–31.5)	26.4 (18.9–33.9)	0.024
Lowest platelet count	150–400 × 10^9^/L	78.5 (4.0–153.0)	82.0 (23.0–141.0)	0.881		68.0 (1.0–135.0)	82.0 (−5.0 to 230.0)	0.339		59.5 (−5.0 to 124.0)	84.0 (17.0–151.0)	0.084
Hepatic function												
Highest AST	3–40 U/L	66.0 (25.5–106.5)	76.0 (38.0–114.0)	0.277		76.0 (42.0–110.0)	75.5 (32.5–118.5)	0.813		81.0 (48.0–114.0)	75.0 (33.0–117.0)	0.909
Highest ALT	3–40 U/L	61.0 (10.0–112.0)	63.0 (9.0–117.0)	0.809		54.0 (8.0–100.0)	63.5 (12.5–114.5)	0.568		49.0 (4.0–102.0)	63.0 (13.0–113.0)	0.441
Renal function												
Highest BUN	7–20 mg/dL	17.0 (4.5–29.5)	24.0 (5.0–43.0)	**0.002**		31.0 (−3.0 to 65.0)	20.0 (5.5–34.5)	0.025		33.0 (0.0–66.0)	20.0 (5.0–35.0)	**0.005**
Highest creatinine	0.5–1.2 mg/dL	1.00 (0.96–1.40)	1.1 (0.3–1.9)	0.091		1.5 (0.7–2.3)	1.0 (0.5–1.5)	**<0.001**		1.5 (0.3–2.7)	1.0 (0.5–1.5)	**0.002**
Lowest GFR	≥60 mL/min	76.0 (37.5–114.5)	67.0 (21.0–113.0)	**0.007**		46.0 (3.0–89.0)	73.0 (33.5–112.5)	**<0.001**		47.0 (1.0–93.0)	73.0 (33.0–113.0)	**0.003**

*Boldface indicates significance. ALT, alanine transaminase; AST, aspartate transaminase; BUN, blood urea nitrogen; GFR, glomerular filtration rate; IQR, interquartile range. †By Wilcoxon 2-sample test.

Six patients underwent lumbar puncture during hospital admission. The indications for lumbar puncture were headache (n = 2), confusion (n = 2), unresponsive state (n = 1), and confusion with bilateral hearing loss (n = 1). None of those patients were found to have a pleocytosis (i.e., all had cerebrospinal fluid [CSF] leukocyte counts <5 cells/µL). One patient had slightly elevated CSF protein (82 g/dL); CSF parameters were otherwise within reference ranges. Five of the 6 patients who underwent lumbar puncture were tested for Lyme antibody in CSF, but results were negative in all cases.

### Risk Factors for Neurologic Complications

Univariate logistic regression models tested associations between comorbid conditions and the 3 most common neurologic symptoms: headache, confusion/delirium, and impaired consciousness. In univariate modeling, we found significant associations (p<0.05) between hypertension, diabetes mellitus, and stroke/transient ischemic attack and the presence of confusion/delirium during hospital admission. Those same factors were also significantly associated with impaired consciousness in univariate analysis and were entered into multivariate models of the respective complications (Table 4).

**Table 4:** Associations between comorbid conditions and neurologic symptoms in patients with babesiosis admitted to Yale-New Haven Hospital, New Haven, Connecticut, USA, January 2011–October 2021*

Comorbid condition	Confusion/delirium, no. (%)	Adjusted odds of confusion/delirium		Impaired consciousness, no. (%)	Adjusted odds of impaired consciousness	
OR (95% CI)	p value	OR (95% CI)	p value
Diabetes mellitus						
No	140 (13.6)	1.00		140 (10.7)		
Yes	23 (34.8)	3.04 (1.11–8.34)	**0.031**	23 (39.1)	5.36 (1.98–14.48)	**<0.001**
Stroke/transient ischemic attack						
No	150 (14.7)					
Yes	13 (38.5)	3.06 (0.88–10.66)	0.079			

*Univariate logistic regression models tested associations between the following comorbid conditions and the 3 most frequent neurologic symptoms (headache, confusion/delirium, impaired consciousness): cardiac disorder, hypertension, diabetes mellitus, immunocompromised status, stroke/transient ischemic attack, obesity, chronic kidney disease, migraine, and malignancy. Variables that were significant in unadjusted univariate analysis were entered into a multivariate model for each neurologic symptom. The significance level for univariate and multivariate analysis was set to p<0.05. Boldface indicates statistical significance.

Multivariate analysis revealed increased adjusted odds of confusion/delirium (OR 3.04 [95% CI 1.11–8.34]; p = 0.031) and impaired consciousness (OR 5.36 [95% CI 1.98–14.48]; p<0.001) among patients with diabetes mellitus. There was a significant association between stroke/transient ischemic attack and confusion/delirium in univariate modeling, but the association was nonsignificant in the multivariate model (p = 0.079). Patients affected by confusion/delirium or impaired consciousness tended to be older (median age 72.5 years [IQR 55.5–89.5]) compared with patients without these symptoms (median age 66.5 years [IQR 46.5–86.5]) (p = 0.012).

### Outcomes

The median length of hospital stay was 6.5 (IQR 2.5–10.5) days for patients with confusion/delirium or impaired consciousness compared with 5 (IQR 2–8) days for those without those symptoms (p = 0.019). A greater percentage of patients with those symptoms were admitted to the intensive care unit (55.3% vs. 44.7%; p = 0.002). Four (2.5%) of the 163 patients in the study group died during hospitalization. Among those 4 patients, 2 had neurologic complications. One experienced ataxia/gait disturbance, confusion/delirium, impaired consciousness, tremor, and vision impairment. The other patient experienced confusion/delirium and impaired consciousness.

## Discussion

More than half (59.5%) of the 163 patients admitted to YNHH with babesiosis had >1 neurologic complication. Confusion/delirium and impaired consciousness were the 2 most common severe neurologic complications among our study patients. Other severe neurologic symptoms, including seizure or stroke, were seldom reported. Confusion/delirium and impaired consciousness were significantly associated with high peak parasitemia (p<0.005) and with markers of renal injury. We also found that the prevalences of confusion/delirium and of impaired consciousness were greater in patients with diabetes mellitus. Although Lyme disease can cause several neurologic complications, including confusion/delirium and impaired consciousness, less than one tenth of patients hospitalized for babesiosis had an erythema migrans rash. We found no relationship between babesiosis–Lyme disease co-infection and prevalence of neurologic complications (*26*–*28*).

The etiology of *Babesia*-associated neurologic symptoms is unknown. Central nervous system complications observed in *B. bovis* in cattle and *B. canis* in dogs are thought to be caused by erythrocyte, platelet, and leukocyte cytoadherence to vascular endothelium with vascular obstruction, excessive proinflammatory cytokine activation associated with high parasitemia, or both (*12*,*29*–*32*). One of the patients in our study had an acute left-middle cerebral artery stroke consistent with vascular obstruction; however, a direct link between stroke and this parasitic infection cannot be established with a single case. Retinopathy has been documented in both cerebral malaria and babesiosis and is thought to be the result of vascular obstruction in the brain and retina (*33*–*35*).

We noted an association between renal impairment (defined by elevated blood urea nitrogen and creatinine and low glomerular filtration rate) and patients who experienced confusion/delirium or impaired consciousness. Confusion/delirium and impaired consciousness may be a direct result of impaired renal function (i.e., secondary to uremia). Alternatively, those neurologic symptoms and renal impairment might be a consequence of a common pathologic mechanism, such as microvascular obstruction from cytoadhering infected erythrocytes or excessive proinflammatory cytokine release. Animal studies likewise have found an association between renal complications and cerebral complications of babesiosis (*31*,*36*–*38*).

Limitations of this study include the retrospective nature of the study design, the lack of histopathologic data, and incomplete access to prehospitalization data, including severity of comorbid conditions and medication use. Our sample size of 163 patients is modest because severe cases of babesiosis requiring hospitalization are uncommon. This study cohort reflects the clinical manifestations of babesiosis patients who experienced severe infection that required hospitalization and, as such, the results may not be directly applicable to all patients with babesiosis. Neurologic symptom severity scores were not available, and duration of symptoms after discharge is unknown, although most patients had cleared or improved neurologic symptoms by the time of discharge. The long-term outcomes of neurologic complications of babesiosis warrant further research.

The lack of histopathologic data limits the specificity of our findings. Patients with severe infections other than babesiosis may also experience many of the neurologic complications described in this study. Regarding the patients we studied, it is challenging to delineate whether signs and symptoms were part of the typical course of systemic infection, were worsened by the presence of babesiosis, or were characteristic of the babesiosis agent itself. Despite those limitations, this study provides a baseline description of the prevalence of neurologic complications of babesiosis.

In conclusion, more than half of the patients admitted to YNHH from January 2011–October 2021 for acute babesiosis experienced >1 neurologic complication. Confusion/delirium and impaired consciousness were each significantly associated with peak parasitemia, renal impairment, and preexisting diabetes mellitus. Further research is needed to clarify the pathogenesis of neurologic manifestations of babesiosis and determine possible long-term neurologic sequelae. Clinicians caring for patients in endemic areas should be aware that babesiosis can manifest with a range of symptoms, including neurologic.

## References

[1] Centers for Disease Control and Prevention. Surveillance for babesiosis—United States, 2019 Annual Summary. Atlanta: The Centers; 2021 [cited 2023 Mar 17]. https://www.cdc.gov/parasites/babesiosis/resources/Surveillance_Babesiosis_US_2019.pdf

[2] Vannier E, Krause PJ. Human babesiosis. N Engl J Med. 2012;366:2397–407. 10.1056/NEJMra1202018 22716978

[3] Herwaldt BL, Linden JV, Bosserman E, Young C, Olkowska D, Wilson M. Transfusion-associated babesiosis in the United States: a description of cases. Ann Intern Med. 2011;155:509–19. 10.7326/0003-4819-155-8-201110180-00362 21893613

[4] Knapp KL, Rice NA. Human coinfection with *Borrelia burgdorferi* and *Babesia microti* in the United States. J Parasitol Res. 2015;2015:587131. 10.1155/2015/587131 26697208 PMC4677215

[5] Diuk-Wasser MA, Vannier E, Krause PJ. Coinfection by *Ixodes* tick-borne pathogens: Ecological, epidemiological, and clinical consequences. Trends Parasitol. 2016;32:30–42. 10.1016/j.pt.2015.09.008 26613664 PMC4713283

[6] Fox LM, Wingerter S, Ahmed A, Arnold A, Chou J, Rhein L, et al. Neonatal babesiosis: case report and review of the literature. Pediatr Infect Dis J. 2006;25:169–73. 10.1097/01.inf.0000195438.09628.b0 16462298

[7] Krause PJ, Gewurz BE, Hill D, Marty FM, Vannier E, Foppa IM, et al. Persistent and relapsing babesiosis in immunocompromised patients. Clin Infect Dis. 2008;46:370–6. 10.1086/525852 18181735

[8] Akel T, Mobarakai N. Hematologic manifestations of babesiosis. Ann Clin Microbiol Antimicrob. 2017;16:6. 10.1186/s12941-017-0179-z 28202022 PMC5310009

[9] Hatcher JC, Greenberg PD, Antique J, Jimenez-Lucho VE. Severe babesiosis in Long Island: review of 34 cases and their complications. Clin Infect Dis. 2001;32:1117–25. 10.1086/319742 11283800

[10] Krause PJ, Auwaerter PG, Bannuru RR, Branda JA, Falck-Ytter YT, Lantos PM, et al. Clinical practice guidelines by the Infectious Diseases Society of America (IDSA): 2020 guideline on diagnosis and management of babesiosis. Clin Infect Dis. 2021;72:185–9. 10.1093/cid/ciab050 33501959

[11] Vannier EG, Diuk-Wasser MA, Ben Mamoun C, Krause PJ. Babesiosis. Infect Dis Clin North Am. 2015;29:357–70. 10.1016/j.idc.2015.02.008 25999229 PMC4458703

[12] Usmani-Brown S, Halperin JJ, Krause PJ. Neurological manifestations of human babesiosis. Handb Clin Neurol. 2013;114:199–203. 10.1016/B978-0-444-53490-3.00014-5 23829910

[13] Bradshaw MJ, Bloch KC. Tick-borne infections of the central nervous system. In: Hasbun R, Bloch KC, Bhimraj MDA, editors. Neurological complications of infectious diseases. Cham: Springer International Publishing; 2021. p. 325–49.

[14] Ortiz JF, Millhouse PW, Morillo Cox Á, Campoverde L, Kaur A, Wirth M, et al. Babesiosis: Appreciating the pathophysiology and diverse sequela of the infection. Cureus. 2020;12:e11085. 10.7759/cureus.11085 33224678 PMC7678756

[15] Sun T, Tenenbaum MJ, Greenspan J, Teichberg S, Wang RT, Degnan T, et al. Morphologic and clinical observations in human infection with *Babesia microti.* J Infect Dis. 1983;148:239–48. 10.1093/infdis/148.2.239 6684141

[16] Venigalla T, Adekayode C, Doreswamy S, Al-Sudani H, Sekhar S. Atypical presentation of babesiosis with neurological manifestations as well as hematological manifestations. Cureus. 2022;14:e26811. 10.7759/cureus.26811 35971375 PMC9374020

[17] Reubush TK II, Cassaday PB, Marsh HJ, Lisker SA, Voorhees DB, Mahoney EB, et al. Human babesiosis on Nantucket Island. Clinical features. Ann Intern Med. 1977;86:6–9. 10.7326/0003-4819-86-1-6 556920

[18] Maxwell SP, Brooks C, McNeely CL, Thomas KC. Neurological pain, psychological symptoms, and diagnostic struggles among patients with tick-borne diseases. Healthcare (Basel). 2022;10:1178. 10.3390/healthcare10071178 35885705 PMC9323096

[19] Oleson CV, Sivalingam JJ, O’Neill BJ, Staas WE Jr. Transverse myelitis secondary to coexistent Lyme disease and babesiosis. J Spinal Cord Med. 2003;26:168–71. 10.1080/10790268.2003.11754578 12828297

[20] Reese M, Maru D. Unexplained recurrent fevers and the importance of inquiring about occupation: a case report. Med Forum. 2016;17. 10.29046/TMF.017.1.012

[21] Krause PJ, McKay K, Thompson CA, Sikand VK, Lentz R, Lepore T, et al.; Deer-Associated Infection Study Group. Disease-specific diagnosis of coinfecting tickborne zoonoses: babesiosis, human granulocytic ehrlichiosis, and Lyme disease. Clin Infect Dis. 2002;34:1184–91. 10.1086/339813 11941544

[22] Walker HK. The origins of the history and physical examination. In: Walker HK, Hall WD, Hurst JW, editors. Clinical methods: the history, physical, and laboratory examinations. Boston: Butterworths; 1990. 21250045

[23] Teasdale G, Jennett B. Assessment of coma and impaired consciousness. A practical scale. Lancet. 1974;2:81–4. 10.1016/S0140-6736(74)91639-0 4136544

[24] Edwards SL. Using the Glasgow Coma Scale: analysis and limitations. Br J Nurs. 2001;10:92–101. 10.12968/bjon.2001.10.2.5391 12170506

[25] Inker LA, Eneanya ND, Coresh J, Tighiouart H, Wang D, Sang Y, et al.; Chronic Kidney Disease Epidemiology Collaboration. New creatinine- and cystatin C-based equations to estimate GFR without race. N Engl J Med. 2021;385:1737–49. 10.1056/NEJMoa2102953 34554658 PMC8822996

[26] Halperin JJ. Nervous system Lyme disease—facts and fallacies. Infect Dis Clin North Am. 2022;36:579–92. 10.1016/j.idc.2022.02.007 36116836

[27] Schwenkenbecher P, Pul R, Wurster U, Conzen J, Pars K, Hartmann H, et al. Common and uncommon neurological manifestations of neuroborreliosis leading to hospitalization. BMC Infect Dis. 2017;17:90. 10.1186/s12879-016-2112-z 28109263 PMC5251276

[28] Mead P. Epidemiology of Lyme disease. Infect Dis Clin North Am. 2022;36:495–521. 10.1016/j.idc.2022.03.004 36116831

[29] Krause PJ, Daily J, Telford SR, Vannier E, Lantos P, Spielman A. Shared features in the pathobiology of babesiosis and malaria. Trends Parasitol. 2007;23:605–10. 10.1016/j.pt.2007.09.005 17988944

[30] Puri A, Bajpai S, Meredith S, Aravind L, Krause PJ, Kumar S. Babesia microti: Pathogen genomics, genetic variability, immunodominant antigens, and pathogenesis. Front Microbiol. 2021;12:697669. 10.3389/fmicb.2021.697669 34539601 PMC8446681

[31] Kumar A, Kabra A, Igarashi I, Krause PJ. Animal models of the immunology and pathogenesis of human babesiosis. Trends Parasitol. 2023;39:38–52. 10.1016/j.pt.2022.11.003 36470781

[32] Allred DR, Al-Khedery B. Antigenic variation and cytoadhesion in *Babesia bovis* and *Plasmodium falciparum*: different logics achieve the same goal. Mol Biochem Parasitol. 2004;134:27–35. 10.1016/j.molbiopara.2003.09.012 14747140

[33] Maude RJ, Beare NA, Abu Sayeed A, Chang CC, Charunwatthana P, Faiz MA, et al. The spectrum of retinopathy in adults with *Plasmodium falciparum* malaria. Trans R Soc Trop Med Hyg. 2009;103:665–71. 10.1016/j.trstmh.2009.03.001 19344925 PMC2700877

[34] Ortiz JM, Eagle RC Jr. Ocular findings in human babesiosis (Nantucket fever). Am J Ophthalmol. 1982;93:307–11. 10.1016/0002-9394(82)90530-X 7200325

[35] Zweifach PH, Shovlin J. Retinal nerve fiber layer infarct in a patient with babesiosis. Am J Ophthalmol. 1991;112:597–8. 10.1016/S0002-9394(14)76867-9 1951603

[36] de Scally MP, Lobetti RG, Reyers F, Humphris D. Are urea and creatinine values reliable indicators of azotaemia in canine babesiosis? J S Afr Vet Assoc. 2004;75:121–4. 10.4102/jsava.v75i3.466 15628803

[37] Aikawa M, Pongponratn E, Tegoshi T, Nakamura K, Nagatake T, Cochrane A, et al. A study on the pathogenesis of human cerebral malaria and cerebral babesiosis. Mem Inst Oswaldo Cruz. 1992;87(Suppl 3):297–301. 10.1590/S0074-02761992000700051 1343706

[38] Tsuji M, Fujioka H, Arai S, Taniyama H, Ishihara C, Aikawa M. A mouse model for cerebral babesiosis. Parasitol Today. 1996;12:203–5. 10.1016/0169-4758(96)40002-3 15275216

